# Multidisciplinary management of hepatocellular carcinoma with portal vein tumor thrombus – Eastern Hepatobiliary Surgical Hospital consensus statement

**DOI:** 10.18632/oncotarget.8386

**Published:** 2016-03-27

**Authors:** Shuqun Cheng, Jiamei Yang, Feng Shen, Weiping Zhou, Yi Wang, Wenming Cong, Guang shun Yang, Hongyan Cheng, Heping Hu, Chunfang Gao, Jia Guo, Aijun Li, Yan Meng, Xiaoqing Jiang, Yefa Yang, Guojun Qian, Ming Luo, Bing Hu, Xiaobo Man, Baohua Zhang, Changqing Su, Feiguo Zhou, Nan Li, Jie Shi, Meng Wang, Yaxin Zheng, Weixing Guo, Juxian Sun, Hongyang Wang, Wan-yee Lau, Meng-chao Wu

**Affiliations:** ^1^ Eastern Hepatobiliary Surgical Hospital, Second Military Medical University, Shanghai, China

**Keywords:** hepatocellular carcinoma, portal vein tumor thrombus, consensus, multidisciplinary treatment, recommendations

## Abstract

Hepatocellular carcinoma (HCC) complicated by portal vein tumor thrombus (PVTT) is associated with poor prognosis, early recurrence of HCC, and limited treatment options. Current guidelines do not have standardized diagnostic and treatment modalities, thus creating a need for a multidisciplinary treatment model for standardization of the treatment. Eastern Hepatobiliary Surgical Hospital (China) convened two working parties of experts from all the departments, to consolidate the current evidence, prevailing vision for the future, and experience of the practicing clinicians engaged in HCC management, so as to develop a consensus for PVTT diagnosis and treatment according to the GRADE system. Based on the quality of the existing evidence and the strength of recommendations, the consensus statements were categorized into 3 evidence levels (A/B/C) and 5 classes (I/II/IIa/IIb/III). The panel discussed and provided clarity on the management and research options in the field of HCC with PVTT. In addition, the panel also assessed the quality of the cited studies and assigned grades to the recommendation statements. Among the group of experts, there was excellent agreement with regard to effective diagnosis and treatment of HCC with PVTT. The recommendations of this consensus will provide guidance to physicians and clinical researchers on the effective management of HCC with PVTT.

## INTRODUCTION

Hepatocellular carcinoma (HCC) is the sixth most common cancer and the third leading cause of cancer-related death worldwide, there has been a continuously increasing trend in the incidence and mortality rates of HCC, with 50% of these being recorded in China alone [1–3]. With advances in diagnosis and treatment for different stages of HCC, the prognosis of HCC patients has been improved. However, there is no substantial increase of the overall survival rate for the past twenty years. Because most cases were still diagnosed at an advanced-stage and the incidence of vascular invasion is quite high. HCC is prone to invading intrahepatic vessels especially the portal vein system. It is reported that the incidence of portal vein tumor thrombus (PVTT) is 44%-62.2% [4], much higher than that of hepatic vein tumor thrombus (HVTT)/inferior vena cava tumor thrombus (IVCTT) and bile duct tumor thrombus (BDT) which is 0.7%-20% and 1.84%-13%, separately [4–6]. PVTT could result in intrahepatic and extrahepatic metastases, portal hypertension, jaundice, and ascites and it was reported to have a median survival time of only 2.7 months. Overall, PVTT is a major risk factor for HCC [7]. Vascular invasion of HCC is divided into macrovascular invasion and microvascular invasion (MVI). Portal vein tumor thrombus (PVTT), which refers to the tumor thrombus in the main portal vein and its branches, is the most common macrovascular invasion and a frequent complication of HCC [4]. MVI is defined as nest-like cancer cells can be seen in vessel lumen covered with endotheliocytes under microscope [7].

Various treatment modalities are available for the treatment of HCC, including surgical resection, liver transplantation, local ablation, transcatheter arterial chemoembolization (TACE), transcatheter arterial radioembolization (TARE), external radiotherapy, and therapy with sorafenib, an oral multikinase inhibitor [8]. However, there is no global consensus on a standardized diagnostic and treatment protocol for PVTT. In 2012, the Europe HCC Guidelines widely accepted the Barcelona Clinic Liver Cancer Staging (BCLC) system as the standard and classified HCC with PVTT as an advance stage (BCLC stage C), recommending sorafenib as the only therapeutic treatment option [9]. But experts in the Southeast Asia hold different opinions. For example, the consensus recommendations by the Asian Pacific Association for the Study of the Liver (APASL) suggested that surgery was listed as a potential radical treatment method for patients of HCC with PVTT [10]. *Diagnosis and Treatment of Primary Liver Cancer (2011)* in China proposed therapeutic options such as sorafenib, surgical resection, radiotherapy, and TACE for the treatment of HCC patients with PVTT [11].

Thus, the management of HCC with PVTT remains unsatisfactory and debatable. Therefore, it is necessary to establish a standardization of diagnosis and treatment for HCC with PVTT in order to ensure optimal patient outcomes, develop scientific categorization, and unify the multidisciplinary understanding of PVTT. In recent years, multidisciplinary treatment (MDT) model combining resection with intra-arterial chemotherapy, radiotherapy, systemic chemotherapy, and/or immunotherapy has received greater attention and recommendation. It has now become imperative to develop MDT for HCC with PVTT. Therefore, Eastern Hepatobiliary Surgical Hospital, the largest centre for HCC treatment in China, convened two working parties of experts from all the departments of the hospital, to consolidate the current evidence, prevailing vision for the future, and experience of the practicing clinicians engaged in HCC management, so as to develop a consensus for PVTT diagnosis and treatment.

## METHODOLOGY

In December 2013, the Shanghai Eastern Hepatobiliary Surgical Hospital established a diagnosis and treatment center for HCC with PVTT in the Second Military Medical University and provided a special diagnosis and treatment channel for PVTT patients. Eastern Hepatobiliary Surgical Hospital convened two great multidisciplinary expert discussions on June 8, 2015, and July 15, 2015, to consolidate the current evidence, persisting view of modern treatments, and relevant MDT experience of practicing clinicians. The group then drafted a document titled *Multidisciplinary Diagnosis and Treatment of Hepatocellular Carcinoma with Portal Vein Tumor Thrombus - Eastern Hepatobiliary Surgical Hospital Consensus* for PVTT patient diagnosis and treatment according to the GRADE system (Table 1). In this document, based on the quality of existing evidence and the strength of recommendations, consensus statements were divided into 3 evidence levels (A/B/C) and 5 classes (I/II/IIa/IIb/III) (Tables 1 and 2). Participants carefully assessed the quality of the cited studies and allotted grades to the consensus statements.

**Table 1:** Recommendation classification

Recommendation Classification	Description
**I**	There is existing supporting evidence or experts tend to believe that this therapeutic measure is beneficial or effective for patients
**II**	There is existing supporting evidence or experts cannot reach consensus on whether a certain therapeutic measure is beneficial or effective for patients
**IIa**	There is existing supporting evidence or experts tend to believe that a certain diagnosis and therapeutic measure is beneficial or effective
**IIb**	There is not enough evidence that a certain therapeutic measure is beneficial or effective, or experts have concerns regarding the validity
**III**	There is existing supporting evidence or experts tend to believe that a certain diagnosis and therapeutic measure is not beneficial or effective or that it may be harmful

A multidisciplinary group of experts from all the departments of the hospital in China − liver surgeons (*n* = 10), radiologists (*n* = 3), pathologist (*n* = 2), radiation oncologist (*n* = 3), oncologists (*n* = 3), hepatologists (*n* = 3) and interventional radiologists (*n* = 4) − convened to develop a consensus for PVTT diagnosis and treatment according to the GRADE system.

First, consensus statements were developed by the corresponding specialists and integrated by academic secretary. Next, we held two seminars to bring the consensus to completion; statements were circulated among the members and modified according to feedback. Upon reaching agreement (≈80%), to accept completely a statement was defined as consensus on that statement. If the consensus was not achieved, the statement was modified and voted upon until consensus was reached. Finally, the group evaluated and recommended each statement's level of evidence.

The recommendation statements were developed and circulated among the members, and modified according to the feedback.

**Table 2:** Strength of recommendations

Evidence Level	Description
**A**	Multicenter and random clinical test or meta-analysis
**B**	Single center's clinical verification or nonrandom research results
**C**	Only from expert opinions, case analysis or diagnosis, and conventional therapy

## CONSENSUS RECOMMENDATIONS

### PVTT occurrence rate and mechanism

PVTT may occur at any stage of HCC, and the occurrence rates are even higher in advanced HCC. It is reported that approximately 10%-40% HCC patients was diagnosed with PVTT when first diagnosed HCC [12]. The incidence was 5.4%-26% among HCC patients who received surgery [13, 14], and 11.3%-38% [15, 16] among non-surgical patients; and 44.0% to 62.2% among autopsy cases [17, 18]. Wu Mengchao et al reported the incidence of PVTT was 6.1% and that of MVI was 67.1% among 5524 HCC patients receiving surgery [19] in Eastern Hepatobiliary Surgical Hospital.

The mechanism of PVTT formation is largely unknown. Existing data suggest PVTT is not only related to abnormal vascular structures inside the tumor, portal venous countercurrent and blood coagulation dysfunction, but also various genes, micro RNAs and abnormal protein expression. It is also believed that changes to the microenvironment caused by HBV infection play an important role. The relation between HBV infection and development of PVTT was validated recently. HBV infection can lead to the elevated TGF-β activity and suppresses the expression of microRNA-34a, resulting in production of chemokine CCL22 which recruits Treg to create an immunesuppresive microenvironment, consequently promoting the PVTT formation [20–24].

The HCC Research Institution of Shanghai Zhongshan Hospital found that arteriovenous shunts of HCC and hepatic lobule structure reconstruction of liver cirrhosis partially provide an experimental basis for the “portal vein countercurrent” theory [25]. PVTT formation may be facilitated by thrombomodulin, which inhibits plasma antithrombase factor and P-selection, through circulating tumor cell adhesion at an early stage. Extensive research has been carried out in the Shanghai Eastern Hepatobiliary Surgical Hospital to elucidate the underlying mechanism of PVTT in HCC. Two HCC cell lines originating from human PVTT, CSQT-1 and CSQT-2, were developed through *in vitro* primary culture, to study the mechanism of development of PVTT [26]. In addition, a microRNA chip was utilized to screen out the differential expression of miR-135a in CSQT-2 and obtain the function of the FOXM1-miR-135a-MTSS1 channel in PVTT formation [27]. The expression of the chemokine receptor CXCR4 was found to be higher in cells from tumor thrombus than paired cancer tissues as well as on CSQT-2, which may explain the development of PVTT in the portal vein [24]. The expression of intercellular adhesion molecule 1 on CSQT-2 cytomembrane has an important role in PVTT formation through the adhesion of mediated hepatoma carcinoma cell, as depicted by real-time polymerase chain reaction and immunoblot analysis on human and mice cell lines [28]. Nonetheless, HCC stem cells and circulating tumor cells are also closely related to PVTT formation.

### Serology marker for PVTT prediction

No specific serum marker for PVTT diagnosis had been established with both satisfactory sensitivity and specificity up to now. However, lately, research in discovering novel molecular predictors for HCC with PVTT has gained attention and a few relevant researches has been conducted to establish some serum markers for HCC accompanied by PVTT [29–36] (Table 3). Other studies have reported some factors related to PVTT occurrence rate such as PIVKA (also known as DCP) [33], AFU, IL8, etc. However, further clinical verification is still required. The diagnosis of PVTT remains challenging and till date there has been no single factor that can perfectly predict PVTT. A combination of multiple methods can increase PVTT predict accuracy; for instance, Shirabe developed a precise scoring system for MVI wherein two of the following three conditions had to be satisfied: tumor diameter >3.6 cm, SUVmax >4.2, or a serum DCP level of 101 mAU/mL, each of these markers had a sensitivity and specificity up to 100% and 90.9%. However, its application in clinical practice is limited [37].

**Table 3:** Overview of Serological Markers for PVTT diagnosis

Reference	Marker	Observation
Liu et al [30]	Serum alpha-fetoprotein (AFP) cancer antigen 125 (CA125)	Serum AFP >32.91 ng/mL and CA125 >113.65 U/mL or AFP >20,000 ng/mLs has reported specificities of 97% and 96%, respectively, for PVTT diagnosis
Kim et al [29]	Thrombus precursor protein (TpP)	Sensitivity and specificity are 82.1% and 3.7%, respectively, for TpP >5.4 ug/mL, indicating that increased D-dimer and TpP levels in HCC are associated with fibrinolysis and coagulation during PVTT and that a positive TpP level is a predictor of tumor thrombosis in HCC
Zhou et al [34]	Plasminogen activator inhibitor (PAI)	Sensitivity is up to 96.0% for PAI, indicating that it is a novel marker for PVTT; however, the specificity is only 38.8 %, thus making its application difficult to apply to clinical practice
Mínguez et al [31]	35-gene signature of vascular invasion	35-gene signature of vascular invasion (14 upregulations and 21 downregulations) has accuracy and negative predictive value of 69% and 77%, respectively, for PVTT diagnosis
Zhuang et al [35, 36]	miR-224 and miR-128-2	miR-224 and miR-128-2 are few among the elevated miRNAs in the serum of HCC patients with PVTT in comparison with HCC patients without PVTT
Pan et al [32]	XAGE-1b	XAGE-1b was significantly correlated with PVTT and tumor-node metastasis (TNM stage) (*P* < 0.05)

### PVTT diagnosis and classification

Simultaneous diagnosis of HCC with PVTT is extremely crucial for improving treatment outcomes and must be commenced if HCC diagnosis is clear and PVTT symptoms exist. Moreover, it is essential to differentiate between malignant and benign PVTT groups for patient prognosis and treatment selection. Although biopsy of a thrombus for histopathological examination is considered the gold standard, PVTT's radiological examinations including contrast-enhanced ultrasound (CEUS), CT scan, MRI's T1WI/T2WI/DWI and enhancement, and DSA have also been used for PVTT diagnosis [38]. While an enhanced scan includes the arterial phase, portal vein phase, and delayed phase, CTA and MRA can comprehensively show the hepatic artery, portal vein, and hepatic vein, thus providing a complete understanding of PVTT. Normalized checking method is the technical assurance for HCC with PVTT. Nevertheless, positron emission tomography/computed tomography (PET/CT) with 2-deoxy-2-18F-fluoroD-glucose (FDG) is of considerable importance in the detection of recurrent HCC with PVTT after treatment [38]. A diagnosis of PVTT can be made if the following radiologic characteristics exist on the basis of HCC diagnosis [39]:

- B-scan ultrasonography reporting that the portal vein is full or partially occupied, mostly with hypoechoic lesions, the color Doppler demonstrates blood flow in the space-occupying lesion
- CT-enhanced scan demonstrating a strip-shaped low-density filling defect shadow in the portal vein in the portal vein stage as well as in the early stage along with a thin line-shaped high-density image in the arterial phase (in some patients)
- MRI showing cavity equisignal or low signal areas in the portal vein space occupying lesion on T1′s weighted image and presence of strip-shape high signal in proton image on T2-weighted image, and filling defect demonstrating similarity with CT
- DSA indicating a line-shaped low-density image parallel to the portal vein, a filling defect with uneven density, or a circular or oval filling defect with a clear boundary
- Presence of a space-occupying lesion in the portal vein, although no tumor metastasis or recurrence is visible in the liver after hepatectomy; HCC postoperative recurrence and tumor thrombus formation should be considered first.

Portal vein thrombosis is usually occured by serious liver cirrhosis or spleen excision or a history of portal vein removal surgery, it should be distinguished it from PVTT. The essentials of PVTT differential points can be summarized as follows:

- Liver cirrhosis Patients with no history of liver cancer or patients who have undergone splenectomy recently and have a surgical history of portal azygos disconnection, with imageological findings of a filling defect within the portal vein suggesting portal vein thrombosis.
- If the patient is diagnosed with HCC and has undergone surgical resection and splenectomy, or small HCC patients (< 5cm) who received radical hepatectomy revealing a portal vein embolism after surgery, it is regarded as portal thrombosis. PVTT is suspected if the embolism does not subside following oral administration of aspirin and other thrombolytic medicines, and an ultrasound-guided fine-needle aspiration biopsy should be performed for differential diagnosis of PVTT.
- If patients have a large HCC(>5cm) or HCC with PVTT before surgery and develop portal vein occupation post-surgery, PVTT should be first considered and should be treated actively as HCC recurrence.
- PVTT generally extends from the portal vein end inside the liver to the extrahepatic area, whereas most cases of portal thrombosis develop from the extrahepatic portal vein to the intrahepatic portal vein branch;
- A few HCC patients have a wide filling defect inside the portal vein and the primary lesion is not obvious.

The location of PVTT is closely related with the prognosis of HCC patients. Currently, TNM staging, BCLC staging, Japan integrated staging (JIS), Cancer of the Liver Italian Program (CLIP), and French scoring system (GRETCH) are the staging systems accepted worldwide that recognize the significance of PVTT [40]. However, they are inadequate because they have not made further refinement or stratification all of which influence analysis and comparison of PVTT patients. The Eastern hepatobiliary classification (also known as Cheng's Classification) and Japan's VP staging classification systems have been introduced for refining HCC staging with PVTT to enable effective prognosis and for guiding surgical treatment (Table 4).

- Japan's PVTT portal vein invasion (VP) classification proposed by the Liver Cancer Study Group of Japan comprises 4 categories VP1, VP2, VP3, and VP4 [8].

The Cheng's Classification comprises 4 categories [41, 42] (Type I, II, III, IV) based on the extent of PVTT invasion on the portal vein where in microscopic portal vein invasion is referred to as Type I_0_. It is noteworthy that this classification combines VP1 and VP2, which are difficult to distinguish and diagnose clinically as Type I. It further subdivides VP4 as Type III/IV, which contains multi-level portal vein, especially emphasizing tumor thrombus inside main trunk (Type III), implicated more treatment selection. The Eastern Hepatobiliary Surgical Hospital [43] analyzed 406 HCC patients with macroscopic PVTT who underwent partial hepatectomy with thrombectomy using Cheng's classification. This retrospective study revealed significant differences for PVTT patients with 1- and 3-year survival rates for type I, II, III, and IV being 52.1%, 38.2%, 24.7%, and 18.3% and 25.1%, 17.7%, 3.6%, and 0%, respectively (*P* < 0.05). This Cheng's Classification is also suitable for HCC patients with PVTT undergoing TACE treatment and reported median survival time of 19.0 months, 11.0 months, 7.1 months, and 4.0 months for type I, II, III, and IV, respectively (*P* < 0.05) [44]. To sum up, the Cheng's Classification better stratified and predicted prognosis than the TNM staging, CLIP scoring system, and JIS scoring system, providing better stratification for evaluating HCC patients.

**Table 4:** Corresponding table of Cheng's Classification and Japan's VP classification

Portal vein	Microscopic PVTT	Segmental branch	Second-order branch	First-order branch	Main trunk	Superior mesenteric vein
Cheng's Classification	I_0_	I		II	III	IV
Japan's VP classification		VP1	VP2	VP3	VP4	

### PVTT treatment options

The principle of treatment is as follows: Based on liver function, removing/controlling PVTT and HCC primary lesion to the best extent possible for better survival. Although sorafenib is the only recommended therapeutic option as per BCLC, experts in China suggest that a combination of multiple methods should be tried depending on patient-specific conditions to achieve the longest possible survival. We should follow the indications of every therapeutic method strictly, and also more high-quality clinical trials should be taken in the future.

### Surgery

#### Surgical resection

Surgical resection is the first choice of treatment for HCC patients with PVTT and is considered a potentially curative strategy. Surgery could remove primary lesion and tumor thrombus simultaneously and is capable of reducing portal venous pressure and improving liver function, quality of life, and survival. Research shows that surgery provides better survival than TACE. Liu et al [45] reported 1-, 3-, and 5-year survival rates of 85% and 60%, 68% and 42%, and 61% and 33% in PVTT patients in the surgical group and TACE group, respectively (*P* < 0.05). Similarly, Peng et al reported that the 1-, 3-, and 5-year survival rates for type I/II PVTT were 42.0%, 14.1%, and 11.1% for the surgical group and 37.8%, 7.3%, and 0.5% for the TACE group, respectively (*P* < 0.001) [46]; however, the effects of the two therapeutic methods are similar for patients with Cheng's type III/IV PVTT. A meta-analysis including 160 HCC patients with PVTT demonstrated that the curative effect of resection was better than TACE treatment [47] for type I/II PVTT; however, no significant differences were found in overall survival between the resection group and the TACE group for patients with type III and type IV PVTT. Therefore, it is widely accepted that surgical resection is suitable for type I/II PVTT with relatively good effect; however, in case of type III/IV the prognosis is extremely poor. At present, the perioperative period mortality rate for HCC patients with PVTT is 0-7.3%, whereas for type I/II PVTT it is < 3%. The five-year overall survival rate for type I/II PVTT and type III/IV PVTT is 10%-59% and 0%-26.4%, respectively.

The indications for hepatectomy are as follows:

- PST grade 0-2; Child-Pugh level A, or according to the hepatic reserve function (ICG15)
- Primary lesion was resectable
- Type I and II PVTT, except extrahepatic metastasis

Based on primary lesion resection, surgical methods for PVTT include thrombectomy with or without peeling off the inner side of the portal vein, and portal vein resection followed by portal vein reconstruction. At present, studies have revealed that there is no obvious difference in prognosis among the three surgical procedures. A surgical margin of less than 1 mm is considered an adverse prognostic factor for long-term survival; however, its significance remains controversial [48]. Kondo et al [49] divided 48 cases of PVTT patients advised surgery into 2 groups based on surgical margins (SM < 1 mm and SM >1 mm). The survival rate in the group with SM >1 mm was longer than the group with SM < 1 mm (497 days vs 227 days, *P* < 0.05). Anatomical hepatectomy under the guidance of 3D imaging can improve resection rates along with radical rescecion, thereby reducing postoperative complications and improving surgical treatment. Eastern Hepatobiliary Surgical Hospital analyzed 74 cases of PVTT patients received surgery (31 cases in the 3D group and 43 cases in the CT group), and found that the operation time of the 3D group was lesser than that of the CT group (167.4 ± 42.6 minutes *vs.* 200.2±71.3 minutes, *P* = 0.026) and the 2-year survival rate was higher than the CT group (40% *vs.* 18%, *P* = 0.023) [50].

The following are certain measures that can help prevent postoperative recurrence:

- TACE after surgery can reduce the postoperative recurrence rate in PVTT patients and extend their survival time (II, C). Peng et al [51] reported that 104 PVTT patients recived surgery; TACE was conducted after surgery in 51 patients and was not conducted in 53 patients who served as the control group. The median survival time was 13 months (95% confidence interval [CI]: 6.25-19.75 months) for the TACE group and 9 months (95% CI: 6.90-11.10 months) for the control group. The estimated 1-, 3-, and 5-year survival rates were higher in the TACE group than in the control group (50.9%, 33.8%, 21.5% *vs* 33.3%, 17.0%, 8.5%, respectively, *P* = 0.0094). Li et al randomly divided 112 PVTT patients advised surgery into the control group (37 cases), postoperative TACE group (35 cases), and postoperative TACE + transportal vein systematic chemotherapy (PVC) group (40 cases) and reported 3-year disease-free survival rates of 17.8%, 23.7%, and 46.1% (*P* < 0.05), respectively [52]. Thus, the disease-free survival rates were higher in the postoperative TACE group and postoperative TACE + PVC group than in the control group.
- Oral administration of sorafenib postoperatively delays HCC recurrence. Eastern Hepatobiliary Surgical Hospital conducted a retrospective study and reported that oral administration of sorafenib post-surgery in PVTT patients can improve the curative effect, with 6-, 12-, 18-, and 24-month survival rates of 61.0%, 38.0%, 13.0%, and 6.0%, respectively [53].
- Oral administration of antiviral drugs can also reduce HCC recurrence rates postoperatively [54] and may be beneficial to PVTT patients.
- Postoperative intravenous chemotherapy and radiotherapy is an area that is yet to be explored.
- The importance of preoperative TACE to prevent postoperative PVTT is still controversial [55], leading to increase chances of risks or may lose the best operation timing.

### Recommendations

- Surgical resection is preferred in patients with PST0-2 grade, resectable HCC primary lesion, liver function Child-Pugh A level, and type I and II PVTT (II,C)
- PVTT patients should have postoperative adjuvant TACE (I, B). PVTT patients should be administered sorafenib orally to reduce the recurrence rate (IIa, C)

### Operative treatment after preoperative radiotherapy and downstaging

Globally, there is still a debate on whether surgical resection is a suitable treatment method for patients with type III PVTT with a resectable HCC primary lesion. Eastern Hepatobiliary Surgical Hospital adopts preoperative radiotherapy and operative treatment only after downstaging for such patients, which was found to be beneficial. This prospective study included 82 HCC surgical patients with main portal vein tumor thrombus (type III PVTT), with 37 patients in the preoperative radiotherapy group with radiotherapy method 3D-CRT and 45 patients in the control group. The result demonstrated that the 2-year survival rate of the preoperative radiotherapy group was apparently higher than the control group (45.9% *vs.* 8.9%, *P* = 0.023); similarly, the 2-year disease-free survival rate was also higher in the preoperative radiotherapy group than in the control group (24.3% *vs.* 0, *P* = 0.000) [56]. The possible advantage of surgical resection after preoperative radiotherapy is that it helps control the progress of the primary lesion and maintains normal liver function; in addition, PVTT downstaging enables radical excision opportunity. It can also reduce recurrence rate, as well as risk during surgery, and postoperative hepatic failure rate [57, 58]. Further research is warranted on preoperative radiotherapy for patients with type I/II PVTT.

The indications for operative treatment after preoperative radiotherapy are as follows:

- PST grade 0-2 and Child-Pugh level A level
- Primary lesions that can be resectable
- Type III PVTT

The contraindications include active peptic ulcer in the alimentary canal, severe esophagus fundus ventriculi varicosity, and extrahepatic metastasis.

Radiotherapy should be given in small doses for a short duration with a split course regimen of 3-5Gy, that is, 6-10 times of irradiation. Operation should be conducted 3-4 weeks after radiotherapy.

### Recommendation

Preoperative radiotherapy should be given in small doses; surgical resection should be performed after 3-4 weeks in patients with PST0-2 grade, resectable HCC primary lesion, liver function Child-Pugh A level, and type III PVTT (IIa, C)

### Non-operative treatment

#### TACE

TACE is the optional treatment for unresectable HCC with PVTT; however, its applicability for type III PVTT is considered controversial owing to the risk of interruption in hepatic arterial blood supply causing hepatic necrosis. Currently, evidence suggests that TACE treatment [59, 60] can be safely provided if patients have good liver function and collateral blood circulation around the obstructed portal vein which has already departed in hepatic hilar region. The mortality rate in the perioperative period is less than 1.2%, and the syndrome occurrence rate after TACE is 28.9%-94%. PVTT treatment with TACE has reported a complete remission rate of 0%, a partial remission rate of 19.5%-26.3%, and a stability rate of 42.5%-62.7%. Luo et al reported patients with type I/II PVTT survived longer than those with type III/IV PVTT (median OS: 10.2 months *vs.* 5.3 months) who received TACE [61]. The indications for TACE are as follows:

- Unresectable HCC primary lesion, type I, II, and III PVTT
- PST 0-2 grade and Child-Pugh of A level
- Presence of collateral circulation open around the main portal vein

It is noteworthy that the therapeutic effect of TACE with lipiodol is superior to transarterial chemoinfusion (TACI) or conservative treatment [47], as reported by a recent meta-analysis evaluating 8 controlled trials. This meta-analysis demonstrated that the 6-month survival rate with TACE was superior to that of TACI (HR: 0.45; 95% CI: 0.2-0.80; *P* = 0.006). TACE significantly improved the 6-month and 1-year survival rates of patients with PVTT compared with conservative treatment (HR, 0.41; 95% CI: 0.32-0.53; *P* = 0.000 and HR: 0.44; 95% CI: 0.34-0.57; *P* = 0.000, respectively). Therefore, the use of granule embolization agents is recommended for PVTT patients with liver function Child-Pugh A level. Lipiodol, gelatin foam, or spring ring are commonly used in China, and literature shows that embolization agents with a smaller diameter have a better effect and lesser adverse effect in PVTT patients. Chern et al, used starch microspheres with a diameter of 47-180 um as embolization agents and reported that the efficacy rate is apparently higher than that in the group with a diameter >180 um (72.2% *vs* 12.5%, *P* < 0.05); however, further clinical verification in China is required [62–64]. Super selective embolization during TACE can reduce normal liver damage and may improve therapeutic effect [59], and there are no special recommendations or specific contraindications for chemotherapeutic agents.

#### Recommendations

- TACE treatment should be preferred in patients with unresectable HCC primary lesion, liver function Child-Pugh A level, and type I and II PVTT (IIa, B)
- TACE treatment (IIb, B) should be used with caution in patients with liver function Child-Pugh B level or HCC patients with type III PVTT (IIa, B)
- TACE treatment should not be used in HCC patients with type IV PVTT (III, C)

#### Radiotherapy

##### External beam radiotherapy

The tolerance dose of whole liver irradiation is only 30-35Gy while it cannot reach the effective tumor exposure dose (35-65Gy). With the technological advancements in radiotherapy, 3D-CRT, IGRT, PBT, and other methods are now available for treatment; it is now possible to deliver high doses of radiation very selectively, with relative sparing of the uninvolved liver. It can be divided into simultaneous irradiation of PVTT and liver tumor or only PVTT irradiation, both of which can be adopted [65]. Previous studies have demonstrated that the total effective rate of radiotherapy is 27.9%-53.8%, while the complete remission rate is 0%-16.7%. The median survival time of patients with effective radiotherapy is 10.7-22 months, whereas only 5-7.2 months in patients with ineffective radiotherapy. Recently, in a retrospective study by Nakazawa et al, radiotherapy demonstrated longer overall survival than sorafenib after performing propensity score matching in patients with PVTT [66].

The indications for external beam radiotherapy are as follows:

- Non-resectable HCC primary lesion, type I, II, III, IV PVTT
- PST 0-2 grade and Child-Pugh level A or B level

The total irradiation amount is 40-65Gy, with a split dose of about 1.8-4.5Gy. Radiotherapy is contraindicated in case of active peptic ulcer, esophagus-fundus vein's severe varix, and extrahepatic metastasis; moreover, precautions should be taken to avoid HBV reactivation during radiotherapy.

Presently, 3D-CRT combined with TACE is mostly adopted in clinical treatment to achieve coordinated anticancer effects. As PVTT receives blood supply from the hepatic artery, TACE may lead to necrosis of tumor thrombus by reducing blood flow. Moreover, TACE will cause shifting of G_O_ stage cell to proliferating phase, thus enhancing radiosensitivity [23, 67]. Literatures in recent years has revealed that the effective rate of 3D-CRT combined with TACE is 39.6%-80.0% and one-year survival rate is 40.0%-58.8%, which was superior to single TACE or radiotherapy. Radiotherapy combined with TACE or hepatic resection can significantly improve the outcomes, but the influence on liver function is smaller for the patients with radiotherapy first than for those with TACE first [68].

#### Recommendations

- Radiotherapy should be conducted in patients with non-resectable HCC primary lesion, liver function Child-Pugh A or B level, and type I, II, III, and IV PVTT (IIa, B*)*
- Combined treatment of radiotherapy and TACE should be considered in patients with liver function Child-Pugh A level and type I, II, and III PVTT (IIa, B)

#### Internal radiotherapy

Frequently adopted internal radiotherapy nuclides include iodine-131 (I^131^), iodine-125 (I^125^), yttrium-90 (Y^90^), and phosphorus-32 (P^32^). Transarterial radioembolization (TARE) is a selective internal radiation therapy delivering tumoricidal radiation with minimal toxicity and alteration in vasculature either through the hepatic artery or by direct implantation in the tumor tissue. Marelli et al [69] analyzed I^131^-lipiodol with TACE and concluded that the average survival rate of the I^131^-lipiodol group is longer than the TACE group (454 days *vs.* 171 days, *P* = 0.025); however, larger studies are still needed to confirm this. Untoward reaction such as whole-body myelosuppression and kidney toxicity must be prevented while using these nuclides. The Y^90^ isotope purely emits β ray with a half-life of 64.2 hours and minimal side effect, showing a curative effect within 10 days. Literature shows that this curative effect is superior to TACE and that it is safer for PVTT patients, with an effective rate up to 28%-50% and median survival time up to 3.2-10.4 months [70]. Lewandowski et al [71] conducted a retrospective analysis of 86 HCC patients with PVTT undergoing either TARE with Y^90^ microspheres or TACE. The partial response rates and overall survival time with TARE were 61% and 35.7 months, respectively, compared with 37% and 15.7 months in the TACE group (*P* < 0.05). Salem et al [72] demonstrated that the response rate and overall survival time of TARE for HCC patients with PVTT were 49% and 20.5 months, respectively, compared with 36% and 17.4 months for the TACE group (*P* < 0.05). Gramenzi et al [73] compared the curative effect of TARE with sorafenib and found comparable overall survival time even after matching for independent prognostic factors including PVTT (13.1 months *vs.* 11.2 months, *P*>0.05). However, no dose criteria for TARE has been specified till date.

#### Recommendation

TARE treatment should be conducted for HCC patients with non-resectable primary lesion, liver function Child-Pugh A level, and type I, II, III PVTT (IIa, B)

#### Sorafenib

Sorafenib is an oral multikinase inhibitor with multiple targets. Literature reveals that ample randomized, double-blind, and parallel control tests have demonstrated sorafenib efficacy in HCC patients [74]. It has already been listed by the CFDA in China as the basic treatment for advanced stage for HCC patients' treatment. Currently, it is the only therapy specifically recommended for HCC with PVTT in the American Association for the Study of Liver Disease (AASLD) and European Association for Study of the Liver (EASL) guidelines. The combination of sorafenib with locoregional therapies including TACE, radiotherapy, and surgery may achieve a better curative effect, but it still remains an area of active investigation. Pan et al [75] reported 170 cases of PVTT patients receiving TACE plus sorafenib treatment and demonstrated a median OS of 13 months (range 1.4-44.8 months) and a median time to progression of 7 months with no untoward reaction. Zhu et al [64] reported that 91 cases of PVTT patients underwent treatment with TACE-sorafenib (*n* = 46) or TACE alone (*n* = 45). Patients with type I and type II PVTT benefited from the combined treatment, with a longer total survival time of 15 months and 13 months, respectively, compared with 10 months (*P* = 0.003) and 6 months (*P* = 0.002) in the TACE-alone group; however, no significant differences were noted in patients with type III PVTT.

#### Recommendation

Oral administration of sorafenib should be used as the basic medicine in PVTT patients either alone or in combination with other treatment methods, e.g., surgery and TACE, etc (I, A)

#### Systematic chemotherapy

Systematic chemotherapy of HCC stagnated for years for lack of effective drugs. With the development of new drugs, many chemotherapeutic agents have been used in the treatment of HCC patients with extrahepatic metastasis, recurrent PVTT, or poor liver function. EACH study in Asia demonstrated that chemotherapy with oxaliplatin has a relatively better curative effect for advanced HCC (including PVTT patients); hence, they are now recommended by physicians in China [76, 77].

#### Recommendation

Systematic chemotherapy is suitable for patients who are contraindicated for surgical resection, TACE, TARE, radiotherapy, etc. or patients with extrahepatic metastasis, or PVTT patients with liver function Child-Pugh A level or B level (IIa, A)

#### Local treatment

At present, local ablation therapies reported in clinical application include percutaneous ethanol injection (PEI), laser ablation (LA), percutaneous radiofrequency ablation (PRFA), and high-intensity focused ultrasound (HIFU). Local ablation can rapidly reduce tumor load and achieve portal blood flow recanalization, but is associated with problems such as damaging the portal vein and bile duct and high rates of PVTT recurrence within a short period [78]. Hence, it is important that the therapy be used with caution and in combination with other methods. Zheng et al [79] reported 134 cases of PVTT patients undergoing RFA with TACE and reported a median survival time of 29.5 months; 1-, 3-, and 5-year survival rates were 63%, 40%, and 23%, respectively, concluding RFA + TACE as an effective therapy. The Eastern Hepatobiliary Surgical Hospital [80] reported 108 cases of PVTT patients with 1-, 2-, and 3-year survival rates of 55.56%, 33.58%, and 22.38% after LA and other comprehensive treatments. Portal vein stent implantation in PVTT patients may cause portal vein blood flow recanalization, thereby increasing hepatic portal vein blood supply; however, it does not reduce the tumor load and should be used in association with other therapies to overcome the same. Vibert et al [81] reported a one-year stent patency rate of 75%, with 1- and 2-year overall survival rates of 47% and 36%, respectively, in 54 PVTT patients undergoing portal vein stent therapy with TACE. Currently, the safety and efficacy of such therapies is under investigation in large randomized trials.

#### MDT work pattern

**Figure 1:**
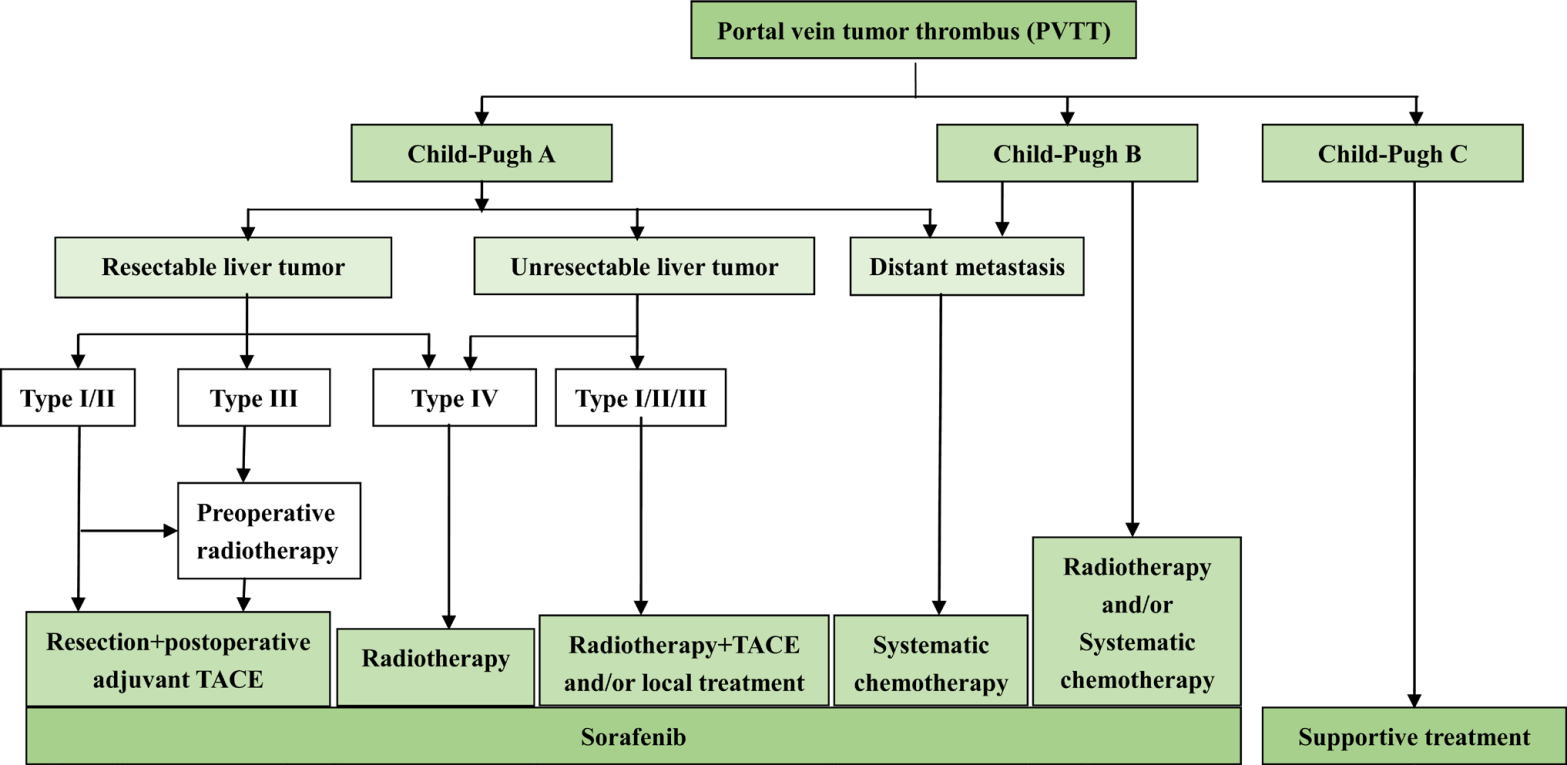
Multidisciplinary Diagnosis and Treatment of Hepatocellular Carcinoma with Portal Vein Tumor Thrombus - Eastern Hepatobiliary Surgical Hospital Consensus: Diagnosis and treatment path diagram

MDT began in the US since the 1990s, and it has become a global trend of disease management. Owing to the absence of a unified treatment standard for HCC with PVTT, MDT may be more adequate. Multidisciplinary coordinated diagnosis and treatment is favorable for maximizing multidisciplinary professional advantages while maximizing patient benefits. Based on existing clinical data of evidence-based medicine, Eastern Hepatobiliary Surgical Hospital formulated a standardized treatment path diagram for HCC patients with PVTT (Figure 1).

## FUTURE PERSPECTIVES

PVTT is a major complication of HCC invasion and metastasis and is the bottleneck of promoting prognosis of HCC. Despite considerable research being conducted on PVTT diagnosis and treatment, there still exist differences of opinion among the Eastern and Western countries. Nonetheless, there is no expert consensus or a standard guideline on the diagnosis and treatment of HCC with PVTT in China. The publication of *Multidisciplinary Diagnosis and Treatment of Hepatocellular Carcinoma With Portal Vein Tumor Thrombus: Eastern Hepatobiliary Surgical Hospital Consensus* will guide clinicians in optimizing therapy outcomes for HCC patients with PVTT. Many issues still need interpretation and verification; An active area of investigation is the development of a scientific and unified stage standard for HCC with PVTT, with a more detailed stage and type analysis on primary tumor progress, liver function Child grade together with the PVTT type, and other multiple indicators. Nevertheless, future recommendations must be based on clear evidence from large, well-controlled clinical trials. Further investigation also should be taken on MDT treatment path, process, and available plan. The relevant molecular mechanism should be elucidated, including PVTT occurrence and development, in order to facilitate more targets for therapies. Microvascular invasion is significant in the prognosis and treatment of PVTT thereby searching for microvascular invasion clinical or biological marker may become an active area of investigation in near future.

## CONCLUSIONS

These recommendations provide guidance on the use of various treatment modalities for better outcomes in HCC patients with PVTT; however, there is scope for further research and summarization in clinical practice and scientific experiment according to new evidence-based medicine.
